# Impact of Processing Approach and Storage Time on Bioactive and Biological Properties of Rocket, Spinach and Watercress Byproducts

**DOI:** 10.3390/foods10102301

**Published:** 2021-09-28

**Authors:** Helena Araújo-Rodrigues, Diva Santos, Débora A. Campos, Suse Guerreiro, Modesta Ratinho, Ivo M. Rodrigues, Manuela E. Pintado

**Affiliations:** 1 CBQF—Centro de Biotecnologia e Química Fina, Laboratório Associado, Escola Superior de Biotecnologia, Universidade Católica Portuguesa, Rua Diogo Botelho 1327, 4169-005 Porto, Portugal; hrodrigues@ucp.pt (H.A.-R.); djesus@ucp.pt (D.S.); dcampos@ucp.pt (D.A.C.); 2 Vitacress Portugal S.A., Quinta dos Cativos, 7630-033 Odemira, Portugal; Suse.Guerreiro@vitacress.com (S.G.); modesta.ratinho@vitacress.com (M.R.); 3 Departamento de Ciências Agrárias e Tecnologias, Escola Superior Agrária, Instituto Politécnico de Coimbra, 3045-601 Coimbra, Portugal; ivorod@esac.pt

**Keywords:** byproducts, rocket leaves, spinach leaves, watercress, freezing and drying impact, antioxidant capacity, polyphenol, carotenoids, vitamin E

## Abstract

The high nutritional value of vegetables is well recognized, but their short shelf life and seasonal nature result in massive losses and wastes. Vegetable’s byproducts are an opportunity to develop value-added ingredients, increasing food system efficiency and environmental sustainability. In the present work, pulps and powders of byproducts from rocket and spinach leaves and watercress were developed and stored for six months under freezing and vacuum conditions, respectively. After processing and storage, microbiological quality, bioactive compounds (polyphenols, carotenoids and tocopherols profiles), antioxidant capacity, and pulps viscosity were analyzed. Generally, the developed vegetable’s pulps and powders were considered microbiologically safe. Although some variations after processing and storage were verified, the antioxidant activity was preserved or improved. A rich phenolic composition was also registered and maintained. During freezing, the quantitative carotenoid profile was significantly improved (mainly in rocket and spinach), while after drying, there was a significant decrease. A positive effect was verified in the vitamin E level. Both processing and storage conditions resulted in products with relevant phenolics, carotenoids and tocopherol levels, contributing to the antioxidant activity registered. Thus, this study demonstrates the potential of vegetable byproducts valorization through developing these functional ingredients bringing economic and environmental value into the food chain.

## 1. Introduction

Vegetables’ and fruits’ short shelf life and seasonal nature result in massive losses and wastes [1,2]. The last report of the Food and Agriculture Organization of the United Nations accounts that in 2016, 21.6% of worldwide fruits and vegetables were lost [3]. In Europe, around 88 million tons of food losses were estimated across all food chains, associated with approximately 143 billion euros [4]. The high quantities of losses and waste generated by vegetable and fruit processing constitute an environmental issue and imply significant costs for food companies [5]. The reduction in these losses is of utmost importance to increase the efficiency of the food system and contribute to environmental sustainability [3,6]. The European Union proposed an action plan for food losses and waste reductions based on a circular economy approach [6]. Vegetables and fruits byproducts such as noncompliant leaves, peels and seeds are rich sources of bioactive compounds, being an opportunity to develop value-added products for the food industry and at the same time combat food losses and waste [5,7,8]. 

In the last years, the consumer demand for healthier products rich in bioactive compounds, minimally processed and without synthetic additives, increased exponentially [7,9,10]. In this context, the green leafy vegetable market gained popularity due to their appealing functional and organoleptic properties coupled with their ready-to-eat availability [9,10,11]. Several studies have reported green leafy vegetables as a rich source of phenolic, carotenoids, α-tocopherol (vitamin E), ascorbic acid (vitamin C) and other bioactive compounds, possessing a higher nutrient value than most of the commonly used vegetables [9,10]. 

Rocket, spinach and watercress are among the most consumed green leafy vegetables, with an exceptional bioactive composition [9]. Several plant food bioactive compounds—for instance tocopherols, carotenoids, phenolic acids and flavonoids—may exhibit antioxidant, antimicrobial, antitumor, anti-inflammatory and other health-related properties [8,9,11,12]. Clinical and epidemiological studies associated with the increasing consumption of vegetables and fruits suggested benefits on cardiovascular, chronic, inflammatory, neurologic and some cancer diseases [1,8,10,12]. The composition and concentration of bioactive compounds present in plant foods depend on numerous intrinsic and extrinsic aspects. Plant genetics, growing and environmental conditions (e.g., water availability, type of soil, light exposure, insect and animal herbivory pressures) and ripening stages are vital parameters. Post-harvest conditions such as transport and storage conditions are also critical factors for the phytochemical profile of vegetables and fruits. In addition, food-processing approaches are one of the most critical parameters that may promote chemical modification or destruction of the natural bioactive metabolites [2,8,9,13]. However, plant foods’ seasonality and perishability require processing and preservation strategies to prolong their shelf-life. Thus, the availability of quality and safe products during off-seasons is guaranteed [2]. 

Freezing is one of the most used processing technology for the long-term preservation of plant foods by industrial companies and households [2,14]. The frozen time and temperature may influence the vegetable and fruit properties due to the biochemical and physical events occurring during freezing storage [14,15]. The ice crystals formed during freezing may damage cellular membranes depending on the location and size, promoting the loss of physical integrity and consequent decompartmentalization of antioxidant compounds. In addition, some authors described another effect: the possible release of bound phenolic acids and anthocyanins and the resultant increase in antioxidant capacity [2,15]. However, although the kinetics of some cellular events, such as the enzymatic and chemical reactions, are slowed during freezing, these are not entirely interrupted [2,14]. Accordingly, reactions occurring during freezing can induce the degradation of bioactive compounds, resulting in a negative impact on the functional properties [2]. 

In contrast, drying is one of the oldest procedures for food processing and preservation, increasing its popularity in the last years [1,8,13]. Their transversal use in the food industry, prolonged shelf-life due to their low water activity, coupled with easy production, storage and transportation, made dry products derived from plant foods very appealing [1,13]. Nowadays, several drying approaches are available, such as hot air, freeze, microwave, infrared and vacuum drying. Although some drying technologies may be most effective and less impactful in the nutritional content of vegetables and fruits, hot air drying is extensively used in the food industry. Hot air drying is typically associated with some phytochemical losses and declines in antioxidant capacity; however, it allows the rapid production of large amounts of dried vegetables with a long shelf-life and fewer costs [1,13]. 

Studies generally lack the understanding of the impact of both processing and storage time on bioactive and biological properties. From a reducing food losses and waste perspective, it is of utmost importance to increase circular economy studies and contribute to the development of functional products [16]. Due to their popularity and health potential, the main goal of this work was to develop pulps and powders from rocket and spinach leaves and watercress byproducts. The biological properties of frozen pulps and powders resultant from hot air drying were evaluated for six months of storage. Microbiological quality and viscosity were monitored. The quantitative profile of key bioactive compounds, such as phenolic compounds, carotenoid and α-tocopherol and related antioxidant capacity, was evaluated during six months of freezing and drying storage.

## 2. Materials and Methods

### 2.1. Chemicals

Sodium hypochlorite (11–15% available chlorine) and HPLC-grade methanol (≥99.9%) were supplied by Honeywell Riedel-de Haën AG, Seelze, Germany. The microbiological media used were purchased from Biokar, namely, plate count agar (PCA), rose bengal chloramphenicol agar (RBCA; Biokar) and Bacillus cereus selective agar (BCSA; Biokar) as well as from VWR specifically, violet red bile glucose agar (VRBGA) and trypticase soy agar (TSA).

Regarding antioxidant and total phenolic compounds (TPC) assays, Folin–Ciocalteu were supplied by Merck (Algés, Portugal), while sodium carbonate, ABTS diammonium salt (2,2′-azino-bis(3-ethylbenzothiazoline-6-sulphonic acid)), 2,2-diphenyl-1-picrylhydrazyl, fluorescein, 2,2′-azo-bis-(2-methylpropionamidine)-dihydrochloride (≥97%; AAPH), butylated hydroxytoluene (≥99%, BHT) and 6-hydroxy-2,5,7,8-tetramethylbroman-2-carboxylic acid (≥97%; Trolox) by Sigma-Aldrich (Sintra, Portugal). Solvents used for HPLC analysis were also purchased from Sigma-Aldrich: acetonitrile, dichloromethane, formic acid, hexane and 2-propanol. Other reagents used during extracts preparation, carotenoids, and vitamin E extraction were also purchased from Sigma-Aldrich (maltodextrin, absolute ethanol, and ascorbic acid). 

Sigma-Aldrich also supplied the chemical standards: β- and α-carotene (≥95%), α-tocopherol (≥95%), β-tocopherol (≥95%), γ-tocopherol (≥95%), δ-tocopherol (≥95%), gallic acid (≥99%), rutin (≥95%), ferulic acid (≥98%), isoferulic acid (≥98%), trans-ferulic acid (≥99%), chlorogenic acid (≥99%), p-coumaric acid (≥98%) quercetin-7-O-glucoside (≥98%), sinapic acid (≥98%), myricetin (≥99%) and caffeic acid (≥98%). The remaining standards were purchased Extrasynthese (Genay Cedex, France): protocatechuic acid (≥99%), lutein (≥95%), lycopene (≥98%), ferulic (≥98%), isoferulic (≥98%), naringenin-7-glycoside (≥99%), naringenin (≥99%), lutein (≥99%) and zeaxanthin (≥98%).

### 2.2. Processing Byproducts

Vitacress Portugal S.A. kindly provided the byproducts present in this study. Rocket and spinach leaves and watercress that did not comply with commercial standards were used. The washing and disinfection step was carried out for 15 min with sodium hypochlorite solution (150 ppm), where a proportion of solution vegetable of 5 L kg^−1^ was applied. The byproducts were rewashed with water abundance and centrifuged to remove wash water. Two different processing methods were used: pulps preparation and subsequent freezing and drying and vacuum storage. 

For pulps development, rocket leaves, spinach leaves and watercress were grounded with a water ratio of 4:1 (vegetable: water), using a vertical slicer blender (Hällde VCB-61) for 2 min. After processing, the pulps were frozen at −20 °C and stored for six months. The sampling points were fresh, M0 corresponded to 24 h after the freezing storage, and M1, M2, M3, M4, M5, M6 corresponding to months one, two, three, four, five and six of the freezing storage, respectively.

Regarding the drying processes, the vegetables were dried in a hot air cabinet, using a 50 °C temperature and 0.65 m s^−1^ of air velocity. The byproducts were then milled and stored for six months under vacuum conditions. In this specific case, the time points were performed every two months due to the increased known stability of dried products. DM0 corresponded to the beginning of the dry experiment (day 0 of storage) and DM2, DM4 and DM6 corresponded to the following sampling times at months two, four and six of the storage period, respectively.

### 2.3. Microbiological Evaluation

Regarding the microbiological analysis of nonwashed, washed vegetables or pulp, 1 g of each fraction was added to 9 mL of sterile 0.1% peptone solution. Each test sample was homogenized in a stomacher (Seward, Worthing, UK) for 1 min. Dilutions were then prepared and spread in the specific culture media. Total aerobic bacteria (TAB) were analyzed according to the international standard (ISO) 4833-1, being this microbial group enumerated in PCA media after incubation for 72 h at 30 °C. Enterobacteriaceae were grown in VRBGA at 37 °C for 24 h. Yeasts and molds were grown and counted on supplemented BCSAB at 30 °C for 3 to 5 days while the Bacillus cereus group was incubated on mannitol egg polymyxin agar at 30 °C for 24 h. 

For main microbial groups enumeration in dry vegetables, the sample homogenization was also performed in a stomacher with 10 g of sample and 90 mL of buffered peptone water. Concerning TAB enumeration, the dilutions were then prepared and spread onto PCA and incubated as previously described. The method described by Ijabadeniyi et al. [17] was followed for both aerobic and anaerobic spore-formers counts. After stomacher homogenization, the stomacher bag was then held at 75 °C for 20 min. After that, dilutional series were prepared and 0.1 mL of each dilution was pipetted into Petri dishes and pour-plated into TSA. Aerobic and anaerobic plates were both incubated at 35 °C for 48 h, but anaerobic plates were placed into anaerobic jars. All analyses were performed in duplicate, the average of microbial counts expressed in log (CFU g^−1^).

### 2.4. Pulps Viscosity Monitorization

To monitor the viscosity of pulps, a rotational springless viscometer B-one plus (Lamy Rheology Instruments, Champagne au Mont d’Or, France) was used, with an R-2 rotor disc (with viscosity values ranging between 200 and 240 M mPa s^−1^) at a speed of 250 rpm for 30 s. The measurements were carried out five times for each sample at room temperature and expressed in mPa s^−1^.

### 2.5. Preparation of Methanolic Extracts

Total phenolic compounds, polyphenolic profile and antioxidant activity were evaluated through the preparation of methanolic extracts. For the preparation of the extracts, 5 g of each homogenized fraction (fresh, frozen pulp, or dry byproduct) were used. The homogenization was carried out with 50 mL of 80% methanol (methanol:water) in an Ultra-turrax^®^ (T18 IKA, Wilmington, DE, USA) at 12,000 rpm for 30 s. The mixture was left under continuous stirring (300 rpm) at room temperature for 2 h. The resulting mixture was then centrifuged at 5000 rpm at 4 °C for 10 min. Then, the supernatant was filtered with Whatman filter paper (no. 1). A rotary evaporator (R-210, Buchi, Switzerland) was used to remove the methanol from the extracts under 40 °C and 175 mbar conditions. The resultant fraction was resuspended in deionized water with 2% of maltodextrin and lyophilized for further analysis. All extracts were prepared in duplicate.

### 2.6. Total Phenolic Content (TPC) Assay

The Folin–Ciocalteau colourimetric method measured the total phenolic content (TPC) present in extracts, as described by Ainsworth et al. [18], with minor alterations. The reaction was performed by mixing 30 µL of extract, 100 µL of Folin–Ciocalteu reagent (20% *v*/*v*) and 100 µL of sodium carbonate (7.4% *m*/*v*) in a 96-well microplate. A standard curve of gallic acid was prepared with concentrations ranging between 0.015 and 0.225 mg/mL, where the same mixture was used, but the standard replaced the extract. The mixture was incubated for 1 h in the dark at room temperature. Subsequently, the absorbance at 750 nm was measured with a multidetection plate reader (Synergy H1, Winooski, Vermont, USA), operated using the Gen5 Biotek software version 3.04. TPC present in extracts was expressed as milligrams of gallic acid equivalent (GAE) per 100 g of dry matter (DM).

### 2.7. Antioxidant Activity

#### 2.7.1. 2,2′-azino-bis(3-ethylbenzothiazoline-6-sulphonic acid (ABTS) Assay

For 2,2′-azino-bis(3-ethylbenzothiazoline-6-sulphonic acid (ABTS) scavenging assay, the method described by Gonçalves et al. [19] was followed, with slight alterations. Firstly, a stock solution was prepared through the reaction of potassium persulfate (2.45 mM) with ABTS^•+^ (7 mM) in ultra-pure water. The reaction was stirred in the dark for 16 h at room temperature. Daily, the stock solution was filtered with a 0.45 µm syringe filter and the absorbance was adjusted to 0.70 ± 0.02 with up water to prepare the ABTS^•+^ working solution. The final reaction volume was 200 µL (20 µL of extract sample and 180 µL of ABTS^•+^ working solution), being the reaction carried out in a 96-well microplate. The control sample consisted of 20 µL of up water and 180 µL of ABTS^•+^ working solution, while in the standard curve (with concentrations between 25 and 175 µM) or sample, the same proportion was used, replacing up water by Trolox or by extract, respectively. The mixture was mixed and incubated in the dark for 5 min. After the reaction period, the absorbance was recorded at 734 nm with also a multidetection plate reader. The extracts were analyzed in triplicate and through the calculation of regression equations, the Trolox concentration in extracts was expressed in mg Trolox equivalent (TE) 100 g^−1^ DM.

#### 2.7.2. Oxygen Radical Absorbance Capacity Assay (ORAC) Assay

The antioxidant capacity was also evaluated by ORAC assay in a multidetection plate reader, as described by Coscueta et al. [20]. Succinctly, in black polystyrene 96-well microplates (Nunc, Denmark), 120 µL of fluorescein (final concentration in the well of 70 nM) and 20 µL of antioxidant extract were mixed in 75 mM of phosphate buffer (pH 7.4). The mixture was pre-incubated at 37 °C for 10 min and 60 µL of APPH solution (final concentration in the well of 12 mM) was then added. In addition to the sample, a blank and calibration standard curve was also added. Phosphate buffer or Trolox were added instead of the antioxidant extract. The incubation occurred for 80 min, being the fluorescence read in intervals of 1 min. The excitation wavelength was set to 485 nm while the emission wavelength to 538 nm. All analyses were performed in duplicate and the results were expressed in mg TE 100 g^−1^ DM.

#### 2.7.3. 2-diphenyl-1-picrylhydrazyl (DPPH) Assay

To quantify the hydrophilic antioxidant compounds, the DPPH assay was performed, following the method described by Schaich et al. [21], with some modifications. A stock solution of DPPH was prepared with a concentration of 600 μM, dissolving 24 mg of DPPH in 100 mL of methanol. A work solution with an approximate concentration of 60 μM was prepared daily by diluting with methanol and adjusting the absorbance to 0.600 ± 0.100 at 515 nm. In the DPPH assay, 25 μL of each extract, standard (Trolox) or negative control (up water) was combined with 175 μL of DPPH working solution in a 96-well microplate. The standard curve ranged between 25 and 175 μM. After the incubation at 25 °C for 30 min in the dark, the absorbance was measured at 515 nm with a multidetection plate reader. All extracts were analyzed in triplicate and Trolox concentration was expressed in mg TE 100 g^−1^ of DM.

### 2.8. Identification and Quantification of Phenolics by High-Performance Liquid Chromatography (HPLC)

Concerning the phenolic profile of methanolic extracts, the method described by Campos et al. [22] was carried out with some alterations. The high-performance liquid chromatography (HPLC) analysis was used, with a chromatographic system with Waters separation module (e2695) and UV/Vis detector (PDA 190–600 nm). A C18 reverse-phase column coupled to a pre-column (Symmetry^®^ C18, Waters, Milford, MA, USA; 100 Å, 5 µm, 4.6 mm × 150 mm) was used. The mobile phases used were phase A (92.5% water: 5% methanol: 2.5% formic acid) and phase B (92.5% methanol: 5% water: 2.5% formic acid), for 59 min of the HPLC run in a continuous flow of 0.5 mL min^−1^. The HPLC conditions started with mobile phase A at 100% and, after 50 min ended with mobile phase B at 55%. Until 55 min, the mobile phase A returns to 100% and remains until the end of the HPLC run. The volume of injection was 50 µL. The detection was performed at wavelengths between 200 and 600 nm with a diode array detector (Waters, Milford, MA, USA), measured at 2 nm intervals. At 280 nm were identified catechins or procyanidins, 320 nm phenolic acids and 350 nm flavonoids by comparing retention time and spectra of pure standards. Each extract replicate was injected in duplicate.

### 2.9. Monitorization of Carotenoid and Vitamin E Content

#### 2.9.1. Carotenoids and Vitamin E Extraction

Total carotenoids and vitamin E isomers (α-tocopherol, β-tocopherol, γ-tocopherol and δ-tocopherol) were extracted according to the method described by Araújo-Rodrigues et al. [16]. Briefly, samples (0.1 g) were mixed with 3 mL of 100% cold ethanol and 0.026 g of ascorbic acid into a screw Teflon-lined cap tube. Saponification was carried out by adding 380 µL of 5 mol L^−1^ KOH (freshly prepared each week, in a solution of 55% absolute ethanol and 45% water), mixing using vortex and shaking water bath (200 rpm) at 85 °C. After 10 min, the tubes were cooled and maintained on ice for the following extraction procedures. Then, 3 mL of 1 mol L^−1^ NaCl was added to the tubes and they were gently mixed. The extraction was performed with 3 mL of 25 μg mL^−1^ BHT solution in n-hexane followed by vigorous vortexing and centrifuging samples (5000 rpm, for 15 min at 4 °C). The organic phase was collected and the extraction was repeated. The new phase was collected again and joined to the first. For each sample, this procedure was performed in triplicate.

#### 2.9.2. Identification and Quantification of Carotenoids by HPLC

The carotenoid profile was identified and quantified according to the method described by Oliveira et al. [23], with slight alterations. The mobile phase used was acetonitrile, methanol, dichloromethane, hexane and ammonium acetate (55:22:11.5:11.5:0.02 *v*/*v*/*v*/*v*/*w*). A Kromasil 100-5-C18 4.6 mm ID, 250 mm column was used at 30 °C. The injection volume was 50 μL of sample extract (in duplicate), the flow rate was constant at 1 mL min^−1^ and the run time was 20 min. The diode-array detector was set at 454 nm to identify the different carotenoids (lutein, β-carotene and α-carotene). The identification was performed by comparing pure standards retention time and the quantification was performed through standard calibration curves, being expressed as mg 100 g^−1^ DM of byproduct.

#### 2.9.3. Identification and Quantification of Vitamin E by HPLC

Tocopherols isomers were analyzed according to the HPLC method described by Savlin et al. [24], with some modifications. Beckman System Gold^®^ coupled to a Waters™ 474 Scanning Fluorescence Detector (excitation and emission wavelengths of 290 and 320 nm, respectively) with a normal-phase silica column (Kromasil 60-5-SIL, 250 mm, 4.6 mm ID, 5 µm particle size) was used. The mobile phase was 1% *v*/*v* isopropanol in n-hexane. Then, 20 µL (in duplicate) was injected, the flow rate was constant at 1 mL min^−1^ and the run time was 20 min. Pure standards of the four tocopherols isomers (α-tocopherol, β-tocopherol, γ-tocopherol and δ-tocopherol) were used to quantify the compounds by using calibration curves. Results were expressed as mg 100 g^−1^ DM.

### 2.10. Statistical Analysis

Statistical analysis was performed recurring to the SPSS statistical software (27.0) via a one-way analysis of variance (ANOVA), at a degree of significance of *p* < 0.05. This statistical analysis was only carried out by analyzing viscosity, TPC, antioxidant capacity, polyphenols, carotenoids and vitamin E. In the microbiological monitoring, only two independent analyses were carried out. In the first phase, data were compared statistically using ANOVA to understand the significance and confirm a normal data distribution. Post hoc multiple comparisons were carried out using Tukey’s test (*p* < 0.05).

## 3. Results and Discussion

In the present study, based on a circular economy approach, rocket and spinach leaves and watercress byproducts were used to study the impact of two processing methods and storage periods on several bioactive properties. Byproducts that do not comply with commercial standards were used to prepare value-added pulps and powders. After the grinding process, the pulps of each vegetable were frozen at −20 °C and all analyses were performed monthly. In parallel, byproducts were also subjected to hot air drying and stored under vacuum conditions. Due to the higher stability of dry byproducts, all analyses were performed every two months, except the monitoring of microbial counts only performed in the initial and final storage period. 

### 3.1. Microbial Counts Evaluation

The main microbial groups were monitored in nonwashed and washed vegetables and pulp and powder forms (Table 1). The analysis of microbial counts in nonwashed byproducts suggested a high microbial load. The TAB counts were approximately 8.20, 8.09 and 8.30 log (CFU g^−1^) in the rocket, spinach and watercress byproducts, respectively. After washing and sodium hypochlorite disinfection, the microbial load of all byproducts significantly decreased. The Portuguese microbiological guidelines classified ready-to-eat vegetables and fruits as satisfactory when the microbial counts of TAB are lower than 6 log (CFU g^−1^), Enterobacteriaceae and yeasts lower than 5 log (CFU g^−1^) and Bacillus cereus spp. lower than 3 log (CFU g^−1^) [25]. Thus, after the washing and disinfection phase, the byproducts in this study were within satisfactory limits, corroborating the importance of this step and the effectiveness of the decontamination agent used. Moreover, in a recent study, where carrot and tomato byproducts were used to prepare pulps and powders, the results suggested a high microbial load in nonwashed vegetables and a significant reduction with the same washing and disinfection step [16]. 

During the freezing period, in the rocket, the TAB counts vary from 4.92 to 5.05 log (CFU g^−1^); in spinach from 4.60 to 4.72 log (CFU g^−1^); and in watercress from 4.94 to 5.00 log (CFU g^−1^) (Table 1). Thus, no considerable variations were registered in TAB microbial counts and the same profile was registered for the other microbial groups monitored. The microbiological data of washed and processed vegetables were within the Portuguese recommended microbiological limits of ready-to-eat products. In the work of Araújo-Rodrigues et al. [16], the microbial monitorization during freezing and drying storages of carrot and tomato byproducts were also evaluated. No key variations in microbial counts during six months of storage were registered. In the near future, if byproducts pulps demonstrate potential through the present study, these can be used in food formulations or for cooking. Nevertheless, the currently produced pulps were within limits for ready-to-eat vegetables and fruits, which is an advantage because no further pre-treatment will be required for food application [16].

Regarding microbial monitorization in dry byproducts, the results globally showed good stability in microbial counts (Table 1), except for TAB and Enterobacteriacea counts in dry rocket byproduct, where a slight decrease was verified. Although the microbial counts agree with those observed for dried spinach and pumpkin leaves by Ntuli et al. [26], it can be considered that these results are relatively high according to Portuguese microbiological guidelines (described above) [25]. Nevertheless, the Portuguese microbiological guidelines only suggest microbial limits for ready-to-eat vegetables and fruits, not including dried products with potential application in food formulations.

### 3.2. Pulps Viscosity Assessment

Pulps viscosity was monitored from fresh samples until M6 of freezing, and the results are presented in Table 2. In all cases, the results suggested a significant decrease in pulps viscosity during freezing, with a more evident decline in viscosity in the first month of freezing. In pulps of rocket and spinach leaves byproducts, significant reductions in pulps viscosity were registered until M5 of storage and after this period, no key variations were found. Regarding watercress byproducts, significant declines were verified in all freezing periods until M6. The ice crystals formed may promote the disruption of the cellular membrane and the loss of water from the intracellular compartments. These events impact physical and physio-chemical properties, for instance viscosity [2,27]. Typical frozen plant foods are associated with loss of textural properties compared to the corresponding fresh forms [2], aligning with the reduction in pulps viscosity registered in the present study.

### 3.3. Monitorization of TPC and Antioxidant Activity

**Figure 1:**
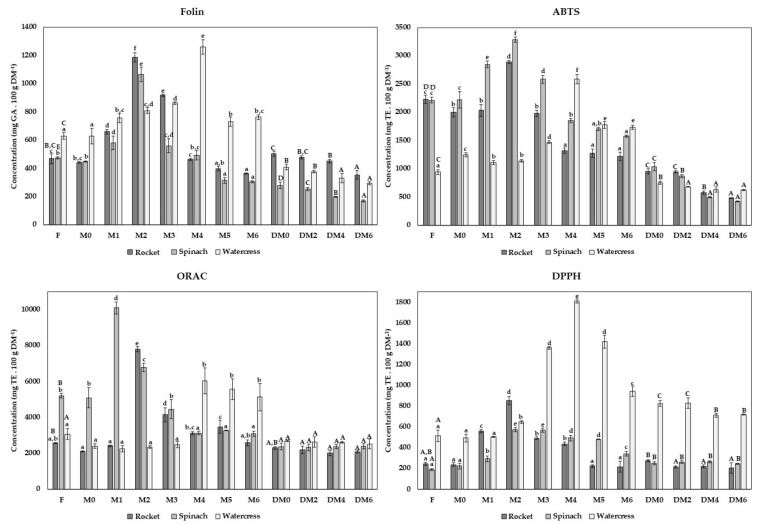
Total phenolic compounds (mg TE 100 g^−1^ DM) and antioxidant capacity (mg GAE 100 g^−1^ DM) by ABTS, ORAC and DPPH assays of the rocket, spinach and watercress byproducts. Total phenolic compounds and antioxidant capacity were evaluated in fresh form (F) and during pulps freezing (M0, M1, M2, M3, M4, M5 and M6, corresponding to 24 h after freezing, months 1, 2, 3, 4, 5 and 6 of freezing, respectively) and drying storage (DM0, DM2, DM4, DM6, corresponding to months 0, 2, 4 and 6 of drying storage). Bars of the same color (same byproduct) and corresponding to the same method of storage (freezing or drying) with different superscript letters are significantly different (*p* < 0.05). Lowercase letters were used for fresh and frozen pulps forms, while uppercase letters were used for fresh and dry powders.

During the application of both approaches and storage periods, TPC and antioxidant activity by ABTS, ORAC and DPPH were monitored through the preparation of methanolic extracts, presented in Figure 1. 

The TPC results of fresh rocket leaves byproducts were 469.51 ± 34.14 mg GAE 100 g^−1^ DM; spinach leaves byproducts were 474.14 ± 8.19 mg GAE 100 g^−1^ DM; and watercress byproducts were 629.60 ± 24.94 mg GAE 100 g^−1^ DM. The results indicated a significant improvement in TPC values during the first months of freezing. Higher TPC values were registered in M2 of rocket and spinach leaves with 1186.65 ± 34.44 and 1065.35 ± 50.68 mg GAE 100 g^−1^ DM, respectively—and in M4 of watercress with TPC value of 1262.13 ± 51.13 mg GAE 100 g^−1^ DM. After this period, the TPC values significantly declined in all frozen byproducts. In the case of the rocket, the TPC values decreased significantly until M6 to 363.10 ± 4.71 mg GAE 100 g^−1^ DM. TPC values of spinach and watercress in M5 and M6 were approximately 310.00 and 740.00 mg GAE 100 g^−1^ DM, respectively, with no key alterations in this final phase of storage. Comparing the TPC results of fresh pulps with pulps at the end of the freezing period, watercress showed slightly higher TPC values in M6, while rocket and spinach presented slightly lower TPC values than fresh forms. The frozen process promotes cell breakages and loss of cellular integrity, which may positively affect TPC and antioxidant capacity due to the decompartmentalization of bioactive compounds. The possible release of bound phenolic acids and anthocyanins during the frozen storage is also suggested by some authors, resulting in the improvement of the functional properties [2,15]. However, these compounds may also be degraded by oxidative enzymes, resulting in a reduction in TPC and antioxidant activity [2]. These events justified the initial increase in TPC values during the first months of freezing and the following reduction in the final phase of the freezing stage.

Concerning spinach and watercress, the TPC values declined significantly to 279.90 ± 21.14 and 409.42 ± 20.13 mg GAE 100 g^−1^ DM during drying, respectively. The TPC values significantly increase during rocket drying until 504.16 ± 17.59 mg GAE 100 g^−1^ DM. During drying storage of all byproducts, the results suggested significant decreases until 354.47 ± 30.33 mg GAE 100 g^−1^ DM in the rocket; 169.96 ± 8.17 mg GAE 100 g^−1^ DM in spinach; and 294.32 ± 11.23 mg GAE 100 g^−1^ DM in watercress in DM6. As previously pointed, several drying technologies are available for vegetables and fruit drying. However, hot air drying is one of the most widely used methodologies at the industrial level, allowing the production of large amounts in a faster and more economical way. However, hot air drying can lead to physical, biological and chemical changes in the overall nutritional profile. These main nutritional variations are associated with the susceptibility of phytochemicals to temperature and consequent significant losses in vitamins, antioxidants, carotenoids and other bioactive compounds [13]. This fact is the primary explanation for the decrease in TPC registered in the present study. 

Different methods were used to evaluate the antioxidant capacity of different time points under study. The ABTS and ORAC assays were used to evaluate the contributions of hydrophilic and amphipathic compounds to the total antioxidant activity. On the other hand, the DPPH assay evaluated the lipophilic compound’s contribution [21]. 

The antioxidant capacity of fresh rocket leaves byproducts was 2228.46 ± 76.95 mg TE 100 g^−1^ DM; spinach leaves byproducts was 2212.57 ± 48.62 mg TE 100 g^−1^ DM; while watercress byproducts was 938.56 ± 41.03 mg TE 100 g^−1^ DM. As suggested by the TPC results, the data of the ABTS assay indicated significant increases in antioxidant capacity during the first months of frozen storage. The highest antioxidant capacity was registered in spinach and rocket in M2, with ABTS values of 3285.07 ± 42.16 and 2889.67 ± 31.52 mg TE 100 g^−1^ DM, respectively. Subsequently, the antioxidant activity decreased until M4 in the rocket (around 1270 mg TE 100 g^−1^ DM), stabilizing until the final storage period. The antioxidant capacity of spinach pulps also decreased after M2, with significant variations until the last storage period, showing an antioxidant capacity of 1575.99 ± 16.22 mg TE 100 g^−1^ DM in M6. Although the spinach and rocket results suggested some decreases in antioxidant capacity in M6 compared to fresh forms, a key improvement in functional characteristics was verified during the initial phase of freezing. Concerning watercress, the highest antioxidant capacity was only registered in M4 (2587.12 ± 82.84 mg TE 100 g^−1^ DM). In M5, a significant decline was verified, but no significant variations were found until M6, with antioxidant capacities around 1750 mg TE 100 g^−1^ DM. Thus, in watercress, the antioxidant capacity by ABTS assay significantly improved with the freezing storage.

In all byproducts, the results suggested that the hot air dry significantly reduced the antioxidant capacity to 1032.69 ± 73.71 mg TE 100 g^−1^ DM in spinach, 952.29 ± 46.99 mg TE 100 g^−1^ DM in rocket and 747.87 ± 29.50 mg TE 100 g^−1^ DM in watercress. This decline was less pronounced in watercress, where some reductions were registered during the first two months of drying storage until approximately 640.45 mg TE 100 g^−1^ DM, with no critical alterations until DM6. Relating to spinach, some key changes were verified until DM4, when the antioxidant capacity was around 457.61 mg TE 100 g^−1^ DM. The results of rocket drying storage suggested significant variations during all storage periods with antioxidant capacity in DM6 of 481.79 ± 3.41 mg TE 100 g^−1^ DM.

Regarding ORAC results of fresh byproducts, spinach leaves byproducts exhibited an antioxidant capacity of 5208.2 ± 130.92 mg TE 100 g^−1^ DM; rocket leaves byproducts of 2565.10 ± 28.38 mg TE 100 g^−1^ DM; and watercress byproducts 3064.98 ± 303.46 mg TE 100 g^−1^ DM. Following the ABTS results, a positive impact of freezing on antioxidant activity was registered, with ORAC values in M1 in spinach (10089.90 ± 325.79 mg TE 100 g^−1^ DM) in M2 in the rocket (7789.00 ± 147.18 mg TE 100 g^−1^ DM) and in M4 in watercress (6038.52 ± 720.05 mg TE 100 g^−1^ DM). In watercress, no significant variations were registered in ORAC antioxidant capacity after M4. After M2, the results indicated key reductions until the final freezing phase but with no significant variations compared with fresh form. The antioxidant capacity of spinach pulps decreased after M1 but no key variations until M4 were verified, with ORAC antioxidant capacity around 3200 mg TE 100 g^−1^ DM.

No key alterations were registered for antioxidant activity determined by ORAC assay in all byproducts studied during all drying storage. No influence of processing was verified for watercress by comparing the fresh and dry results. In spinach, significant reductions were verified after the drying process, with an antioxidant capacity of 2379.74 ± 191.26 mg TE 100 g^−1^ DM in DM0. In contrast, only a slight decline in antioxidant capacity was verified in the rocket compared to fresh leaves byproducts.

Although both ABTS and ORAC assays measure the contribution of hydrophilic and amphipathic compounds to antioxidant capacity, the variations between antioxidant capacities found resulted from the distinct reaction mechanisms underlying. In the case of ABTS assay, the radical used possesses a higher molecular weight than the transfer mechanism molecule of hydrogen atom used in ORAC assay. Thus, in ABTS a steric block of active centers may occur, resulting in a decrease in its reaction rate. As suggested by Campos et al., this reason makes this method considered more accurate [22]. This fact coupled with using a radical biological source in ORAC assay makes this a more relevant technique [28].

As previously stated, the DPPH method evaluates the contribution of lipophilic compounds to the total antioxidant capacity, requiring the use of organic solvents [21]. Generally, the DPPH values of fresh byproducts were lower than the antioxidant capacity determined by the other methods. In fresh forms, rocket leaves byproducts, spinach leaves byproducts and watercress byproducts had an antioxidant capacity of 245.06 ± 16.75, 190.79 ± 9.40 and 515.02 ± 53.57 mg TE 100 g^−1^ DM, respectively. The same increasing profile followed by a significant decline was registered in DPPH assay, with the higher antioxidant values in rocket and spinach in M2 (853.65 ± 41.04 and 575.28 ± 19.42 mg TE 100 g^−1^ DM, respectively) and in watercress in M4 (1811.05 ± 18.14 mg TE 100 g^−1^ DM). An extremely positive effect in antioxidant capacity was registered in watercress; even with a significant reduction after M4, it showed higher values than the fresh byproduct, namely, 943.08 ± 48.21 mg TE 100 g^−1^ DM. Contrary to the other antioxidant assays, the results suggested that watercress byproducts possessed considerably higher DPPH values than other byproducts during both freezing and drying storage. A possible explanation to these results is the high presence of isothiocyanates in watercress. These very reactive compounds are hydrophobic [29] and consequently measured by DPPH assay. Moreover, a positive impact was verified in spinach pulps freezing with an antioxidant capacity of 341.84 ± 20.97 mg TE 100 g^−1^ DM, higher than fresh form. Concerning rocket byproducts, no significant variations were found compared to the fresh byproduct and M6 frozen pulp. 

During spinach drying, a significant improvement was registered in DPPH antioxidant activity until 251.19 ± 18.85 mg TE 100 g^−1^ DM, with no significant variations during all storage phase. The same tendency was suggested (DM0: 274.97 ± 10.36 mg TE 100 g^−1^ DM) in rocket, but with a significant reduction until 215.52 ± 18.85 mg TE 100 g^−1^ DM in DM2, stabilizing in the remaining storage period. Finally, in watercress, the results indicated a considerable increase in antioxidant capacity after drying, with DPPH values of 825.50 ± 29.71 mg TE 100 g^−1^ DM in DM0 and a decline until approximately 714.00 mg TE 100 g^−1^ DM in DM4, coupled with maintenance until the final period of storage. Thus, spinach and watercress showed higher antioxidant capacity values in all drying storage period rocket than fresh byproducts, showing a positive impact of hot air drying in DPPH antioxidant capacity. In this study, lipophilic compounds such as carotenoids and tocopherols were quantified (see Section 3.5). A significant increase in carotenoids and tocopherols in the powders (compared to fresh form) was verified, especially in watercress. These results aligned and justified the notable increase in antioxidant activity with this method for watercress.

Several variations in TPC and antioxidant activity values are found in the literature resultant from intrinsic and extrinsic parameters such as vegetable variety, cultivation and environmental conditions (e.g., light exposure, water availability), germination and maturation stages as well as post-harvest factors, for instance, storage conditions, processing technologies [2,9,30,31,32] and methodologies used for extracts preparation [9]. Intrinsic factors are suggested as a determinant for the metabolism of phenolic compounds [9]. In the present study, the whole vegetable byproduct in the case of watercress was used for processing, while in the case of spinach and rocket, only leaves byproducts were used. Thus, the use of whole vegetables or just the leaves for processing may also justify the differences reported in the literature for fresh byproducts.

As explained in the discussion of the TPC results, the increase in antioxidant capacity in the first months of frozen storage could be promoted by the loss of cellular membrane integrity, resulting in bioactive compounds release and an increase in antioxidant activity [2,15]. To this fact, the possible release of bound phenolic acids and anthocyanins may be coupled. Nevertheless, after this initial improvement, the reduction in antioxidant capacity may be explained by chemical and enzymatic reactions that induce the degradation of bioactive compounds [2]. The literature data agree with these results, although few studies were found under the same processing and freezing conditions in the literature for the studied byproducts. Kamiloglu [33] also studied TPC changes in spinach samples during industrial freezing, suggesting an excellent nutritional value during freezing storage and aligning with the present study data. As previously reported, after the increase in the TPC and antioxidant capacity stage, some losses were registered compared to the fresh forms with M6 in spinach, but a high nutritional value was registered. The watercress and rocket results also aligned with this report, where a high nutritional value was registered during all experimental procedures. In the case of watercress, higher TPC and antioxidant capacity values were verified in M6 comparatively with fresh forms, while in the rocket, the results suggested some losses.

Most of the literature data found using blanching before freezing to inactivate enzymes involved in deterioration during frozen storage. Thus, no literature studies were found to directly compare the present results during freezing storage. For example, other authors studied the effect of spinach freezing but with the previous application of blanching, showing significant reductions in TPC and ORAC antioxidant capacity compared with fresh spinach. The authors suggested that this considerable decrease may be associated with the mild heat treatment used in the blanching process [32]. Ninfali et al. [34] also studied the effect of spinach freezing on antioxidant capacity, and the results suggested that blanching and freezing decreased the antioxidant capacity by the ORAC method. Moreover, Puupponen-Pimiã et al. studied the effect of blanching and freezing in spinach, but no variations were detected in the antioxidant capacity by DPPH assay. Other authors also reported losses in polyphenols in watercress during blanching/freezing [35].

Generally, the literature data focus on the impact of green leafy vegetables drying on TPC and antioxidant activity, aligned with the present study results. As suggested by the work of Pickmony et al. [13], using a hot air dryer significantly decreased TPC and antioxidant activity of watercress. In the reported study, the authors studied the impact of different drying temperatures, namely, 40, 50 and 70 °C, under constant air velocity of 1.20 m s^−1^ on some nutritional parameters of watercress leaves. The results suggested that all nutritional parameters evaluated were negatively correlated with the increase of drying temperature, including TPC and ABTS antioxidant activity. Other work studied the effect of convective oven dry at 65–70 °C on spinach leaves and stems’ TPC and antioxidant capacity. The results suggested decreased TPC and antioxidant capacity by ORAC and DPPH assays by dry weight after dehydration [36], aligning with the present study results. As previously mentioned, hot air drying is one of the most used strategies for vegetable and fruit drying across the industrial chain. However, the temperature can promote losses in antioxidant capacity and TPC [13]. 

### 3.4. Phenolic Composition

**Figure 2:**
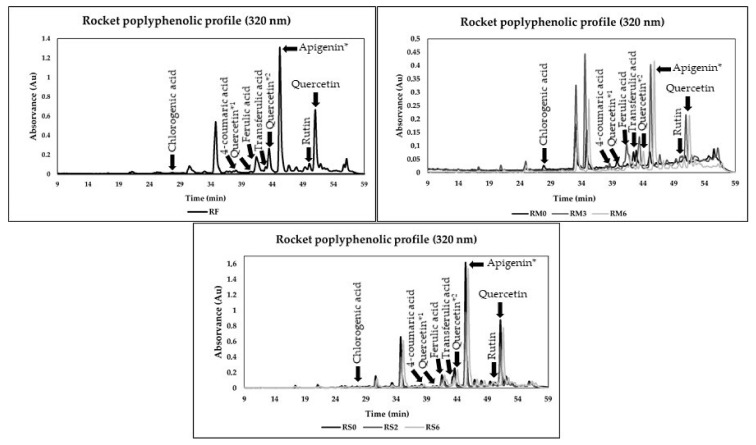
Chromatograms of free phenolic compounds identified of fresh rocket leaves byproduct (RF) and during the pulps freezing (RM0, RM3, RM6, corresponding to 24 h after freezing and month 3 and 6 of freezing, respectively) and drying storage (DM0, DM2, DM6, corresponding to month 0, 2 and 6 of drying storage) at a wavelength of 320 nm. * = derivative.

Polyphenolic chromatograms of fresh rocket byproducts, during pulps freezing and dry storage, are present in Figure 2. The quantitative analysis of polyphenolic compounds suggested a rich composition with some alterations after applying processing strategies and during storage time. Different phenolic acids belonging mainly to hydroxycinnamic acid, flavonols and flavones families were identified.

**Figure 3:**
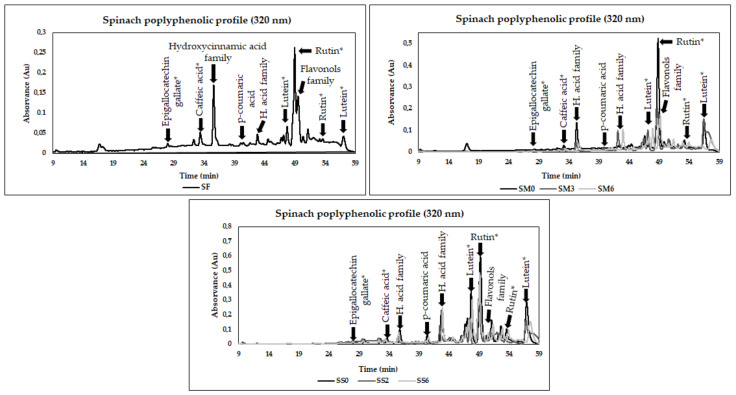
Chromatograms of free phenolic compounds identified of fresh spinach leaves byproduct (SF) and during the pulps freezing (SM0, SM3, SM6, corresponding to 24 h after freezing and month 3 and 6 of freezing, respectively) and drying storage (SM0, SM2, SM6, corresponding to month 0, 2 and 6 of drying storage) at a wavelength of 320 nm. * = derivative.

The polyphenolic chromatograms of fresh, freezing and drying spinach byproducts are represented in Figure 3, where phenolic compounds of hydroxycinnamic acid, flavonols, flavones and flavanols groups were the antioxidant compounds detected. Variations in the polyphenolic profile were registered after processing strategies used and during the storage period.

**Figure 4:**
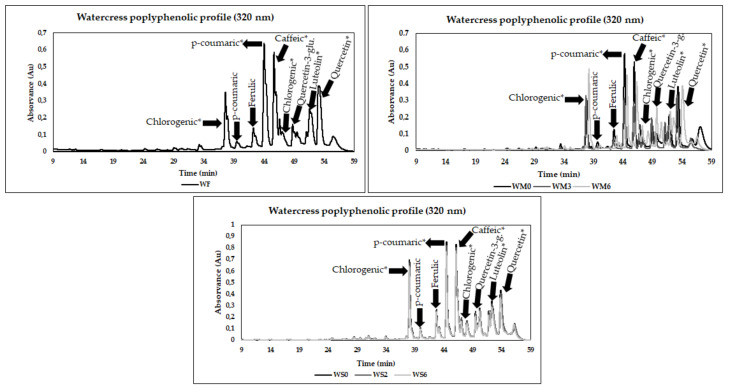
Chromatograms of free phenolic compounds identified of fresh watercress byproduct (RF) and during the pulps freezing (RM0, RM3, RM6, corresponding to 24 h after freezing and month 3 and 6 of freezing, respectively) and drying storage (DM0, DM2, DM6, corresponding to month 0, 2 and 6 of drying storage) at a wavelength of 320 nm. * = derivative.

The chromatograms of polyphenols of watercress byproducts are present in Figure 4. In fresh byproducts, frozen pulps or powders storage, the hydroxycinnamic acids, flavonols and flavones were identified as the most prevalent phenolic antioxidants. The results also suggested a high diverse profile and some variations after pulps freezing and drying processing and during storage.

Quantitative profiles of polyphenolic compounds present in rocket and spinach leaves and watercress byproducts during all experimental periods were also determined by HPLC and are summarized in Table 3. The results suggested significant variations in the polyphenolic compounds identified during the freezing and drying storage for rocket byproducts. Phenolic antioxidants belonging to flavonols (quercetin-3-glucoside and quercetin derivatives), flavones (apigenin and rutin) and hydroxycinnamic acid (chlorogenic, p-coumaric, ferulic and transferulic acids) families were identified and quantified. Apigenin derivative was the most abundant antioxidant in fresh rocket leaves, frozen pulps and powders. A high presence of quercetin and quercetin derivatives and compounds of the hydroxycinnamic acid family were also verified. Previous work investigated the phenolic profile of different fresh baby-leaf vegetables, including wild rocket, spinach and watercress [9]. In the reported study, several quercetin derivatives were quantified in the fresh wild rocket. The most prevalent groups identified were hydroxycinnamic acids and flavonols. Another study investigated the phenolic profile of different genetic sources of a wild and cultivated rocket, with quercetin derivatives as the most prevalent phenolic group detected [37]. Li et al. [38] studied the phenolic profile of several cruciferous vegetables. The rocket results also suggested an interesting phenolic composition with several hydroxycinnamic acids and flavonoids detected. For instance, ferulic acid, rutin, quercetin-3-glucoside and several quercetin derivatives were also identified and quantified in the reported study, aligning with the present study.

During freezing storage, some significant variations were observed. Chlorogenic, p-coumaric, ferulic and transferulic acids, apigenin, rutin and quercetin-3-glucoside declined significantly after freezing. However, after this period, the concentration of these phenolic compounds increased significantly, followed by a new decrease phase. In chlorogenic and transferulic acids and rutin, this period was followed by a new increase and decrease. Concerning quercetin derivatives, the freezing improved the concentration of these antioxidant compounds, followed by a final stage of concentration decrease. Some losses were reported compared fresh forms with M6, mainly in the case of chlorogenic, p-coumaric, ferulic and transferulic acids and rutin and quercetin-3-glucoside. However, a positive effect of freezing was also verified in quercetin derivatives and apigenin concentration. Thus, the results suggested that spinach pulps possessed a very interesting polyphenolic profile during freezing and after six months of storage.

The drying process significantly decreased the chlorogenic acid and rutin concentrations, but no significant variations during storage were verified. No significant variations in p-coumaric concentration were verified after the drying process and during storage. In ferulic acid, no key variations were registered with processing; however, a significant decline in its concentration was visible after DM4. In contrast, the concentration of quercetin and apigenin derivatives increased significantly after drying, while the concentration of transferulic acid and quercetin-3-glucoside declined significantly. The monitorization of these antioxidant compounds suggested some significant decreases during drying storage. The antioxidants variations during freezing and drying processes were previously discussed in the “Monitorization of TPC and antioxidant activity” section.

The main phenolic compounds detected in spinach were from hydroxycinnamic acid (*p*-coumaric acid and caffeic acid derivative), flavones (luteolin and rutin derivatives) and flavanols (epigallocatechin gallate derivative) families. The most prevalent antioxidant was luteolin derivative in fresh, frozen pulps and dry byproducts. Other works focusing on fresh spinach’s phenolic profile also suggested the hydroxycinnamic acid group as the most prevalent phenolic group in spinach [9,39,40]. In contrast, Kamiloglu [33] suggested flavonoids and phenolic acid (e.g., p-coumaric acid derivatives) as predominant polyphenols.

The results suggested significant reductions in phenolic compounds in M0, namely epigallocatechin gallate and caffeic acid derivatives. During freezing storage, the concentration of these antioxidants increased significantly until M6. In caffeic acid derivative, a significant reduction was verified from M4 to M5, followed by a new improvement until M6. However, in both cases, the concentration was significantly higher than in fresh spinach. Regarding *p*-coumaric acid, it was not detected in fresh form; however, during pulps freezing, its concentration increased significantly with a higher concentration in M4, decreasing drastically until the final storage period (where it was also not detected). In the remaining phenolic compounds detected, the results indicated a significant increase, with the maximum concentration in M4 in the case of lutein derivative 1, while other lutin derivative and rutin derivatives in M4 decreased significantly in all cases until M6. However, in all cases, a significant improvement of these compounds was verified comparatively with fresh forms. Kamiloglu [33] studied the impact of industrial freezing on spinach polyphenols, suggesting that freezing can be an excellent strategy to preserve these bioactive groups and aligning with the present study results. 

The drying processing negatively affected caffeic acid derivatives, with no variations registered during the storage period. On the other hand, after drying processing, the results suggested a positive impact in *p*-coumaric acid and derivatives of epigallocatechin gallate, rutin, and luteolin. No variations were verified during storage in luteolin derivative 1. Some declines were detected in epigallocatechin gallate derivative, *p*-coumaric, luteolin derivative 2 and rutin derivatives. However, key improvements comparatively with fresh spinach byproducts were registered, suggesting that the drying approach may be a good approach to improve the spinach polyphenolic profile.

Concerning watercress byproducts, the results suggested a higher concentration of phenolic compounds in all forms analyzed. The most prevalent was the hydroxycinnamic acid group (chlorogenic acid derivative, caffeic acid derivative, ferulic acid, *p*-coumaric acid and derivatives). Recently, some authors also studied the phenolic profile of fresh watercress, being flavonols and hydroxycinnamic acid groups the most representative [9]. Moreover, quercetin derivatives, as well as *p*-coumaric acid, caffeic acid and caffeic acid derivatives, were detected by these authors. As previously mentioned, other work focused on the phenolic composition of 12 cruciferous vegetables, including rocket and watercress. This study suggested hydroxycinnamic acid as the most incident group in watercress, followed by the flavonoids group [38]. The reported study identified and quantified caffeic, ferulic and *p*-coumaric acids, quercetin-3-glucoside, quercetin and luteolin derivatives.

The pulps processing and freezing negatively impacted chlorogenic, *p*-coumaric and caffeic acid derivatives and quercetin derivative, with significant reductions after frozen storage (24 h of freezing). Regarding *p*-coumaric acid and ferulic acid, no key variations were registered with processing. In contrast, the quercetin-3-glucoside and luteolin derivative were improved with the preparation of pulps and freezing processing, where significant increases were registered. During freezing storage, a first period with some significant declines was registered in the quercetin derivative, quercetin-3-glucoside, *p*-coumaric acid derivatives and ferulic acid until M3. In the chlorogenic acid derivative, *p*-coumaric acid, caffeic acid and luteolin derivatives, significant declines were verified until M4. After this period, the results suggested a significant improvement until the final stage of freezing. *p*-coumaric acid derivatives, quercetin-3-glucoside and quercetin derivatives showed higher concentrations in M6 than fresh forms, indicating a positive effect of freezing storage in the concentration of these antioxidants. 

The drying processing negatively affected the chlorogenic acid derivative, *p*-coumaric acid, ferulic acid, *p*-coumaric derivatives and quercetin derivatives, but no significant variations were registered during all storage periods. The caffeic acid and luteolin derivatives results also suggested a significant decline after drying, with minor variations during the storage period. On the contrary, quercetin-3-glucoside concentration increased after the drying process, and some variations during storage were also verified. Accordingly, the results of phenolic compounds identified and quantified in watercress suggest that freezing may be the most effective way to preserve or enhance phenolic compounds in watercress byproducts. 

Generally, the results aligned with TPC results obtained by Folin–Ciocalteu. However, some variations between HPLC quantifications and TPC results were registered, mainly due to the detection limit of phenolic antioxidants. Other reason may be the interferent molecules existent in prepared extracts (e.g., proteins and sugars). These can react with Folin–Ciocalteu reagent, resulting in overestimation [33,41]. Table 4 lists all phenolic antioxidants identified and quantified in the rocket, spinach and watercress byproducts and their retention times and maximum absorption wavelengths (λ max).

### 3.5. Carotenoids and Vitamine E Content

**Figure 5:**
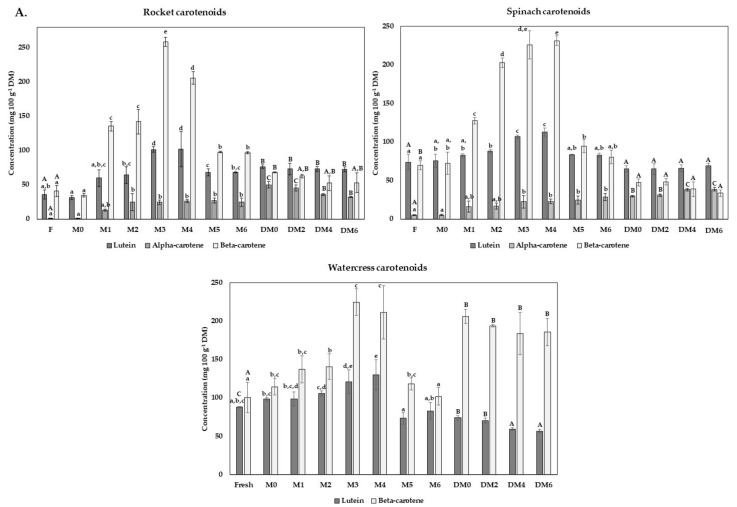

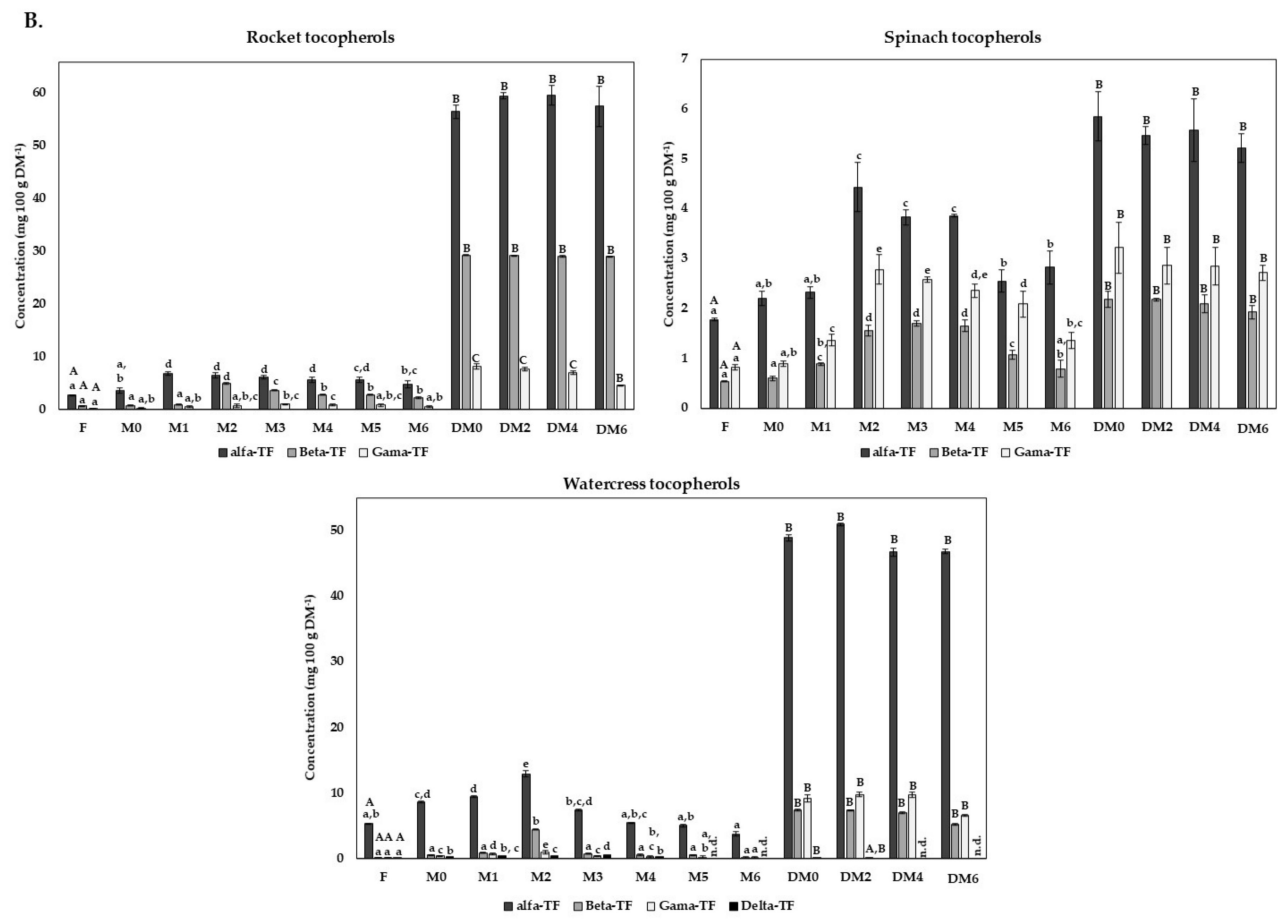
Concentration (mg 100 g^−1^ DM) of carotenoids (**A**) and vitamin E (**B**) detected in the rocket, spinach and watercress byproducts. Carotenoids and tocopherols (TF) concentrations were evaluated in fresh form (F) and during pulps freezing (M0, M1, M2, M3, M4, M5 and M6, corresponding to 24 h after freezing, months 1, 2, 3, 4, 5 and 6 of freezing, respectively) and drying storage (DM0, DM2, DM4, DM6, corresponding to months 0, 2, 4 and 6 of drying storage). Bars of the same colour (same carotenoid or TF isomer) and corresponding to the same method of storage with different superscript letters are significantly different (*p* < 0.05). n.d = not detected.

The carotenoid profile was also monitored and is represented in Figure 5. α-, β-carotene and lutein were detected in spinach and rocket leaves byproducts. In contrast, in watercress byproducts, only β-carotene and lutein were identified. The most relevant carotenoid in all vegetables was β-carotene and lutein with concentrations of 40.66 ± 8.12 and 36.09 ± 6.68 mg 100 g^−1^ DM in the fresh rocket, 69.75 ± 5.66 and 74.03 ± 9.91 mg 100 g^−1^ DM in the fresh spinach, and 100.74 ± 19.67 and 87.86 ± 1.21 mg 100 g^−1^ DM in the fresh watercress. These results confirmed previous results, which also found lutein and β-carotene as the main carotenoids in leaf vegetables [42,43,44]. Previous works on rocket’s carotenoid content verified that different levels of total glucosinolate content presented different carotenoid profiles. The major carotenoids were lutein and β-cryptoxanthin in some samples, and others were lutein, β-carotene and zeaxanthin [45]. This fact could explain some differences among the results of this study and others in the literature. Another study with watercress demonstrated that nitrogen concentrations in nutrient solutions along plant growth affect carotenoid content differently. Lutein had a higher response to the nitrogen concentration than β-carotene, which means that higher nitrogen concentration increases lutein content to a greater extent than β-carotene [46]. Moreover, genotypes affect carotenoids content, as a study with spinach from different genotypes demonstrated [47].

In all byproducts, a significant increase in carotenoid content was registered during the freezing period. The higher concentrations were registered in rocket and watercress in M3 at 258.54 ± 6.56 and 224.89 ± 17.22 mg 100 g^−1^ DM, respectively, while in spinach in M4 at a concentration of 231.55 ± 5.92 mg 100 g^−1^ DM. After this period, the β-carotene concentration decreased, with some significant variations. In the case of the rocket, a significant decrease was registered after M3 with no key variations after M5, with β-carotene concentrations around 97.00 mg 100 g^−1^ DM. Regarding watercress, after M3, the β-carotene concentrations reduced considerably until 118.24 ± 7.74 mg 100 g^−1^ DM in M5 and until 102.06 ± 11.49 mg 100 g^−1^ DM in M6. After M4 of freezing, the results indicated that β-carotene in spinach pulps declined drastically in M5, with a slight decrease until 80.56 ± 9.12 mg 100 g^−1^ DM in M6. Lutein content followed the same tendency, with the higher values in M4 in all byproducts, decreasing until approximately 68.00, 83.00 and 78.22 mg 100 g^−1^ DM of the rocket, spinach and watercress, respectively, with no key alterations from M5 to M6. Although in lower concentrations, α-carotene concentration increased during all freezing period in spinach, with no significant variations from the M3. In the rocket, α-carotene concentration improved until M5, with no key concentration changes from M2 until the final phase of freezing storage. Previous works also demonstrated an increased carotenoids content after freezing [48]. The increase in the carotenoids’ content after freezing was possibly related to the freezing damaged induced through the ice crystals produced into cell membranes. Depending on the size and location, thawing leads to carotenoids release from the cell wall [2]. The carotenoid content decreases after few months of storage, being justified by chemical and enzymatic reactions that naturally occur through freezing over time, causing carotenoid degradation once they are in the free form [2,14]. To the author’s knowledge, no data from the literature was available to directly compare the carotenoids and tocopherols profile of the present data during freezing storage.

During drying of rocket leaves, the α-, β-carotene and lutein concentrations significantly increase compared with fresh form, with concentrations of 50.03 ± 5.02, 68.16 ± 0.78 and 76.47 ± 2.68 mg 100 g^−1^ DM, respectively. Previous studies have demonstrated that the milling process promotes the liberation of antioxidant molecules associated with fiber, which could explain the increase in carotenoids content because the free form of the carotenoids is evaluated in samples [49,50]. The results suggested that lutein becomes the most predominant carotenoid in the dry rocket, with no significant variations during drying storage. Some declines in α- and β-carotene were verified in DM4 and DM2, respectively. After releasing carotenoids into the free form, it is expected that their content decreased due to their vulnerability during storage by isomerization and degradation by chemical and enzymatic reactions [2,51]. However, in both cases, higher concentrations were found during all storage phases compared with fresh forms. The drying of spinach leaves did not impact the lutein concentrations, and also, no changes were verified during storage. α-carotene concentration increased with drying (DM0: 29.72 ± 1.02 mg 100 g^−1^ DM), with a slight increase in DM2, while a negative effect in β-carotene concentration was verified (DM0: 47.38 ± 4.50 mg 100 g^−1^ DM), with no variations during six months of storage. Finally, the watercress drying process had a positive impact on β-carotene concentration, where its concentration was improved until 206.18 ± 9.24 mg 100 g^−1^ DM. In contrast, drying may negatively impact lutein concentrations, which showed approximately 72.00 mg 100 g^−1^ DM in DM0 and DM2 and after DM4 concentrations around 58.07 mg 100 g^−1^ DM.

α-tocopherol, β-tocopherol, γ-tocopherol and δ-tocopherol (isomers of vitamin E) content was also assessed during all experiments (Figure 5). These bioactive compounds are lipid-soluble compounds with antioxidant capacity and are essential in the human diet [52].

All four tocopherols were present in the watercress, but in both rocket and spinach vegetables, only the α-, β- and γ-tocopherol were detected. For all vegetables, α-tocopherol was the major tocopherol found in fresh byproducts, pulps and powders. Fresh rocket presented an α-tocopherol content of 2.68 ± 0.08 mg 100 g^−1^ DM. β-tocopherol content of 0.64 ± 0.02 mg 100 g^−1^ DM, and the γ-tocopherol content was 0.22 ± 0.01 mg 100 g^−1^ DM. Fresh spinach presented a content of α-, β- and γ-tocopherols of 1.78 ± 0.03, 0.54 ± 0.01 and 0.82 ± 0.05 mg 100 g^−1^ DM, respectively. Watercress tocopherols content were 5.31 ± 1.49, 0.20 ± 0.08, 0.10 ± 0.02 and 0.11 ± 0.05 mg 100 g^−1^ DM for α-, β-, γ- and δ-tocopherols, respectively. Other works found the α-, and γ-tocopherol in the rocket, spinach and watercress where α-tocopherol was in higher content than the γ-tocopherol [43,53,54,55]. The values of tocopherols content found in this study have the same magnitude order of other literature studies. Previous studies demonstrated that the differences in the tocopherols’ content values could be due to plant genotype biostimulant and irrigation treatment [54].

Except for spinach, the pulps elaboration induced liberation of tocopherols, significantly increasing their concentration (Figure 5). This fact is explained by the fresh-cut processing effect on cell wall structures that causes the liberation of bound bioactive compounds [51]. As previously described for the carotenoid content, the freezing process also impacted the tocopherols content, which is mainly associated with the ice crystals generated upon the freezing process that disrupts cell membrane releases bounded compounds to the fiber during thawing [51]. During freezing storage of rocket pulps, there is an increase in α-tocopherol content until M1 (6.80 ± 0.29 mg 100 g^−1^ DM), with no significant variations until M5. After this period, a decline in the α-tocopherol concentration from 5.64 ± 0.53 to 4.74 ± 0.70 mg 100 g^−1^ DM was verified. A continuous increase in β-tocopherol until M2 was also registered (4.94 ± 0.32 mg 100 g^−1^ DM) and a significant decrease until M3 was verified (3.64 ± 0.24 mg 100 g^−1^ DM), followed by stabilization until M6. No key variations were seen in γ-tocopherol content in the rocket, ranging its concentration between 0.28 ± 0.11 and 0.99 ± 0.05 mg 100 g^−1^ DM. During freezing storage of spinach pulps, there is an increase in tocopherol content until M2, in the case of α-tocopherol and γ-tocopherol, namely 4.40 ± 0.49 and 2.79 ± 0.30 mg 100 g^−1^ DM, while in the case of β-tocopherol, until M3 (1.70 ± 0.05 mg 100 g^−1^ DM). Posteriorly, α-tocopherol concentration in spinach declines significantly until M5 and then stabilizes, with concentrations around 2.69 mg 100 g^−1^ DM. Concerning β-tocopherol and γ-tocopherol concentrations in spinach, the concentration decreases significantly until M6, namely 0.79 ± 0.16 and 1.36 ± 0.16 mg 100 g^−1^ DM, respectively. In watercress pulps, α, β- and γ-tocopherols content increases until M2 of storage (α-tocopherol: 12.92 ± 1.58 mg 100 g^−1^ DM; β-tocopherol 4.47 ± 3.43 mg 100 g^−1^ DM; and γ-tocopherol 0.95 ± 0.02 mg 100 g^−1^ DM) and δ-tocopherol until M3 (0.63 ± 0.09 mg 100 g^−1^ DM), but start decreasing after this period. β-tocopherol and α-tocopherol concentrations decline significantly until M3 (0.76 ± 0.12 and 7.37 ± 1.45 mg 100 g^−1^ DM, respectively), in the first case stabilizing until the final phase of storage. In contrast, in the α-tocopherol profile, minor variations were verified. Concerning γ-tocopherol concentration, the results suggested a decline until 0.08 ± 0.02 mg 100 g^−1^ DM (M6). Finally, δ-tocopherol concentration in the watercress decline until 0.30 ± 0.02 mg 100 g^−1^ DM (M4), and after this period, it was not detected. 

The powder’s elaboration significantly increased the tocopherols content to a more extended amount than carotenoids. Previous works demonstrated that tocopherols are more stable than carotenoids, especially β-carotene [44]. Rocket powder contained 56.59 ± 1.27, 29.32 ± 1.27 and 8.14 ± 0.24 mg 100 g^−1^ DM of α-, β- and γ-tocopherols, respectively (DM0), while spinach powders presented contents of α-, β- and γ-tocopherols of 5.86 ± 0.50, 2.18 ± 0.16 and 3.22 ± 0.52 mg 100 g^−1^ DM, respectively. Regarding watercress powders, these had tocopherols contents of 48.98 ± 2.76, 7.45 ± 1.07, 9.21 ± 1.44 and 0.18 ± 0.01 mg 100 g^−1^ DM for α-, β-, γ- and δ-tocopherols, respectively. The increase of these compounds in powders was also associated with the same reasons explained by carotenoids increase. Milling induces liberation of bioactive compounds bounded to fiber [49,50]. Generally, no key variations were registered during six months of storage on the tocopherols content of all the vegetable powders, which shows great stability of these compounds under this storage condition. Some exceptions were verified in rocket γ-tocopherol, where a significant decrease was verified in DM6 until 4.54 ± 0.39 mg 100 g^−1^ DM. The results also indicated a decline in watercress δ-tocopherol concentration during dry storage, and from DM4, this isomer was not detected. As previously appointed for carotenoids, some chemical and enzymatic reactions can lead to some degradations after releasing tocopherols into the free form [2,51].

## 4. Conclusions

The present work suggested that the green leafy vegetables, namely rocket and spinach leaves and watercress byproducts, can be valorized by processing into two types of food ingredients: pulps and powders. The results corroborated that these added-value ingredients are rich in bioactive compounds such as phenolic compounds, carotenoids and vitamin E, and presented great antioxidant activity. The TPC and antioxidant capacity typically increase during the first months of freezing and decrease after this period. Although some losses were registered, the frozen pulps of rocket and spinach generally showed values close to the fresh byproduct, presenting a high antioxidant capacity after six months of freezing storage. The results suggested a higher TPC and antioxidant capacity in M6 than in fresh form in watercress pulp. The TPC and antioxidant capacity in the powders were often lower than fresh forms but still demonstrating a high TPC and antioxidant capacity. On the contrary, the antioxidant capacity of watercress evaluated by the DPPH assay suggested a significant increase with drying, while no significant variations observed by ORAC assay.

Concerning phenolic composition, the results indicated a rich composition, where bioactive compounds belonging to hydroxycinnamic acid, flavonols, flavones and flavanols families were identified and quantified in the three byproducts studied. Some variations after processing and storage were verified depending on the antioxidant metabolite, with some improvements and declines. However, generally, a rich phenolic composition was found during all storage periods. The carotenoid profile was significantly improved with freezing (mainly in rocket and spinach), while a more significant positive impact was verified in the vitamin E profile during drying. However, freezing and drying processing, followed by storage, resulted in products with very interesting carotenoid and tocopherol profiles.

Generally, both ingredients were considered safe in microbial counts and pre-served great antioxidant activity and bioactive compounds during storage or, in the case of watercress, improved the functional profile of fresh byproducts. Thus, this study demonstrates the high economic and environmental potential of recovery of these functional ingredients into the food chain.

## Figures and Tables

**Table 1:** Microbial counts (*n* = 2) expressed in log(CFU g^−1^) of main microbial groups present in rocket, spinach and watercress byproducts. The microbial counts were evaluated before and after the washing process and during six months of pulps freezing and drying storage.

Vegetables	Sample	TAB	*Enterobacteriacea*	Yeasts and Molds	*Bacillus* *cereus* spp.	Aerobic Spore-Forms	Anaerobic Spore-Forms
Rocket	Non washed	8.20 ± 0.18	7.00 ± 0.07	5.95 ± 0.10	4.80 ± 0.09	-	-
	Washed	4.95 ± 0.10	3.81 ± 0.08	3.70 ± 0.09	2.84 ± 0.10	-	-
	Fresh pulp	5.04 ± 0.03	3.92 ± 0.03	3.75 ± 0.15	2.92 ± 0.05	-	-
	M0	5.00 ± 0.09	3.84 ± 0.08	3.89 ± 0.13	2.90 ± 0.03	-	-
	M1	5.02 ± 0.09	3.85 ± 00.08	3.82 ± 0.11	2.83 ± 0.13	-	-
	M2	5.05 ± 0.10	3.89 ± 0.02	3.74 ± 0.29	2.70 ± 0.05	-	-
	M3	5.02 ± 0.04	3.86 ± 0.05	3.73 ± 0.03	2.67 ± 0.09	-	-
	M4	4.92 ± 0.04	3.84 ± 0.15	3.66 ± 0.11	2.71 ± 0.08	-	-
	M5	4.99 ± 0.10	3.81 ± 0.09	3.71 ± 0.15	2.69 ± 0.15	-	-
	M6	4.96 ± 0.14	3.84 ± 0.03	3.69 ± 0.08	2.72 ± 0.08	-	-
	DM0	6.07 ± 0.84	6.16 ± 0.17	-	-	4.95 ± 0.61	3.92 ± 0.11
	DM6	4.99 ± 0.13	4.08 ± 0.13	-	-	4.47 ± 0.92	3.52 ± 0.74
Spinach	Non washed	8.09 ± 0.09	6.58 ± 0.04	5.59 ± 0.05	4.43 ± 0.05	-	-
	Washed	4.54 ± 0.10	3.98 ± 0.05	3.58 ± 0.13	2.49 ± 0.09	-	-
	Fresh pulp	4.62 ± 0.09	4.03 ± 0.04	3.61 ± 0.16	2.51 ± 0.16	-	-
	M0	4.65 ± 0.10	4.01 ± 0.10	3.59 ± 0.14	2.56 ± 0.10	-	-
	M1	4.67 ± 0.11	4.00 ± 0.08	3.61 ± 0.19	2.55 ± 0.14	-	-
	M2	4.67 ± 0.05	4.03 ± 0.07	3.67 ± 0.05	2.58 ± 0.13	-	-
	M3	4.68 ± 0.16	4.04 ± 0.11	3.75 ± 0.09	2.61 ± 0.17	-	-
	M4	4.60 ± 0.20	4.02 ± 0.11	3.73 ± 0.05	2.69 ± 0.02	-	-
	M5	4.72 ± 0.18	4.04 ± 0.09	3.76 ± 0.06	2.64 ± 0.15	-	-
	M6	4.65 ± 0.16	4.02 ± 0.15	3.68 ± 0.03	2.66 ± 0.05	-	-
	DM0	>5.48 ± 0.00	5.46 ± 0.49	-	-	5.85 ± 0.27	4.74 ± 0.06
	DM6	6.93 ± 0.49	6.31 ± 0.56	-	-	5.33 ± 0.22	4.48 ± 0.43
Watercress	Non washed	8.30 ± 0.18	7.23 ± 0.11	6.67 ± 0.08	4.45 ± 0.05	-	-
	Washed	4.80 ± 0.15	4.06 ± 0.06	3.82 ± 0.12	2.51 ± 0.11	-	-
	Fresh pulp	4.96 ± 0.10	4.12 ± 0.13	3.87 ± 0.16	2.74 ± 0.11	-	-
	M0	4.99 ± 0.10	4.14 ± 0.12	3.81 ± 0.18	2.69 ± 0.17	-	-
	M1	4.94 ± 0.18	4.11 ± 0.06	3.82 ± 0.10	2.72 ± 0.16	-	-
	M2	4.96 ± 0.15	4.10 ± 0.04	3.87 ± 0.16	2.77 ± 0.09	-	-
	M3	4.98 ± 0.06	4.12 ± 0.06	3.88 ± 0.11	2.67 ± 0.23	-	-
	M4	5.00 ± 0.05	4.14 ± 0.08	3.91 ± 0.10	2.75 ± 0.16	-	-
	M5	4.96 ± 0.05	4.17 ± 0.06	3.85 ± 0.13	2.64 ± 0.08	-	-
	M6	4.94 ± 0.07	4.11 ± 0.08	3.86 ± 0.13	2.65 ± 0.14	-	-
	DM0	>6.48 ± 0.00	7.00 ± 0.05	-	-	6.15 ± 0.35	5.24 ± 0.29
	DM6	7.49 ± 0.53	6.42 ± 0.08	-	-	6.06 ± 0.01	5.14 ± 1.04

**Table 2:** Rocket, spinach and watercress pulps viscosity (mPa s^−1^) from fresh pulps to six months of freezing storage.

Vegetable	Sample	Viscosity (mPa s^−1^)
Rocket	Fresh pulp	364.24 ± 3.22 ^g^
	M0	311.02 ± 3.93 ^f^
	M1	299.12 ± 2.79 ^e^
	M2	290.76 ± 1.97 ^d^
	M3	282.68 ± 0.81 ^c^
	M4	273.20 ± 2.46 ^b^
	M5	264.41 ± 3.85 ^a^
	M6	257.00 ± 1.93 ^a^
Spinach	Fresh pulp	155.70 ± 2.12 ^f^
	M0	121.24 ± 1.10 ^e^
	M1	115.18 ± 1.71 ^d^
	M2	107.10 ± 0.90 ^c^
	M3	103.66 ± 0.89 ^b,c^
	M4	100.23 ± 3.60 ^b^
	M5	96.22 ± 1.99 ^a^
	M6	92.46 ± 1.63 ^a^
Watercress	Fresh pulp	253.68 ± 7.97 ^h^
	M0	203.82 ± 1.69 ^g^
	M1	194.42 ± 3.54 ^f^
	M2	180.78 ± 1.99 ^e^
	M3	169.22 ± 1.17 ^d^
	M4	152.12 ± 3.40 ^c^
	M5	140.80 ± 1.07 ^b^
	M6	135.80 ± 3.96 ^a^

Columns corresponding to the same byproduct with different superscript letters are significantly different (*p* < 0.05).

**Table 3:** Concentration (mean ± standard deviation) of phenolic compounds (mg 100 g^−1^ DM) present in the rocket (R), spinach (S) and watercress (W) byproducts (n = 4). Phenolic compounds concentration at wavelengths of 280, 320 and 350 nm were evaluated in fresh form (F) and during pulps freezing (M0, M1, M2, M3, M4, M5 and M6, corresponding to 24 h after freezing, months 1, 2, 3, 4, 5 and 6 of freezing, respectively) and drying storage (DM0, DM2, DM4, DM6, corresponding to month 0, 2, 4 and 6 of drying storage).

**R**	**Chlorogenic Acid**	***p*** **-Coumaric Acid**	Quercetin *^1^	**Ferulic** **Acid**	**Transferulic Acid**	Quercetin *^2^	Apigenin *	**Rutin**	**Quercetin-3-glucoside**
F	6.0 ± 0.47 ^d,B^	1.3 ± 0.6 ^c,d,e,A^	4.1 ± 0.1 ^a,A^	6.0 ± 0.1 ^c,B^	15.8 ± 0.3 ^f,C^	12.2 ± 0.4 ^a,A^	137.31 ± 0.74 ^a,A^	36.4 ± 0.3 ^h,B^	48.3 ± 1.2 ^f,C^
M0	1.3 ± 0.03 ^a,b^	0.6 ± 0.0 ^a,b^	5.6 ± 0.8 ^a^	3.4 ± 0.1 ^a^	4.6 ± 0.0 ^e^	19.1 ± 1.5 ^b^	88.17 ± 0.41 ^b^	15.6 ± 0.2 ^f^	38.9 ± 3.8 ^e^
M1	1.5 ± 0.09 ^a^	1.7 ± 0.3 ^e^	15.2 ± 0.1 ^c^	5.1 ± 0.0 ^b^	2.4 ± 0.1 ^c^	49.7 ± 0.4 ^d^	105.81 ± 1.66 ^c^	12.3 ± 0.9 ^e^	22.9 ± 2.5 ^b^
M2	7.5 ± 1.09 ^e^	3.3 ± 0.3 ^f^	48.7 ± 1.8 ^g^	12.7 ± 0.1 ^e^	0.8 ± 0.0 ^a^	131.3 ± 4.8 ^g^	102.49 ± 0.77 ^c^	2.3 ± 0.2 ^a^	27.2 ± 1.5 ^c^
M3	2.9 ± 0.10 ^c^	1.6 ± 0.1 ^d,e^	31.8 ± 0.8 ^f^	25.2 ± 0.4 ^g^	2.3 ± 0.2 ^b,c^	65.8 ± 3.2 ^e^	205.04 ± 2.53 ^d^	6.4 ± 0.6 ^c^	18.1 ± 1.5 ^b^
M4	2.0 ± 0.00 ^a,b,c^	0.9 ± 0.1 ^a,b,c^	17.2 ± 0.3 ^d^	17.3 ± 0.0 ^f^	1.9 ± 0.2 ^b^	87.4 ± 1.8 ^f^	232.71 ± 4.76 ^e^	22.9 ± 1.1 ^g^	34.3 ± 1.8 ^d^
M5	2.6 ± 0.03 ^b,c^	1.0 ± 0.1 ^b,c,d^	24.3 ± 0.1 ^e^	6.7 ± 0.4 ^d^	3.8 ± 0.3 ^d^	34.7 ± 0.3 ^c^	314.35 ± 0.19 ^f^	8.2 ± 0.5 ^d^	20.5 ± 0.6 ^b^
M6	1.3 ± 0.00 ^a^	0.4 ± 0.0 ^a^	11.6 ± 0.0 ^b^	3.2 ± 0.0 ^a^	1.9 ± 0.0 ^b,c^	16.5 ± 0.0 ^a,b^	149.82 ± 1.22 ^g^	4.0 ± 0.0 ^b^	9.8 ± 0.0 ^a^
DM0	2.3 ± 0.07 ^A^	1.6 ± 0.1 ^A^	31.7 ± 0.9 ^C^	5.5 ± 0.4 ^B^	3.1 ± 0.0 ^B^	45.9 ± 0.5 ^D^	444.75 ± 5.23 ^B^	14.0 ± 0.9 ^A^	14.9 ± 0.5 ^B^
DM2	2.1 ± 0.00 ^A^	1.4 ± 0.1 ^A^	29.1 ± 1.7 ^C^	5.5 ± 0.1 ^B^	2.9 ± 0.1 ^B^	39.5 ± 1.1 ^C^	429.89 ± 3.24 ^C^	13.6 ± 0.6 ^A^	14.1 ± 0.1 ^A,B^
DM4	2.1 ± 0.01 ^A^	1.5 ± 0.1 ^A^	22.0 ± 0.0 ^B^	4.2 ± 0.0 ^A^	3.0 ± 0.1 ^A^	34.4 ± 0.1 ^B^	400.60 ± 0.43 ^D^	13.4 ± 0.9 ^A^	13.8 ± 0.2 ^A,B^
DM6	2.0 ± 0.16 ^A^	1.5 ± 0.0 ^A^	22.3 ± 1.3 ^B^	4.4 ± 0.3 ^A^	2.0 ± 0.3 ^A^	35.6 ± 1.4 ^B^	407.05 ± 1.35 ^D^	13.7 ± 0.8 ^A^	12.4 ± 0.8 ^A^
**S**	Epigallocatechin Gallate *	Caffeic Acid *	***p*** **-Coumaric Acid**	Lutein *^1^	Rutin *^1^	Rutin *^2^	Lutein *^2^	-	-
F	10.9 ± 0.1 ^b,A^	8.5 ± 0.7 ^d,B^	N.D.	4.3 ± 0.0 ^a,A^	2.4 ± 0.1 ^a,A^	2.7 ± 0.0 ^a,A^	21.2 ± 0.2 ^b,A^	-	-
M0	3.1 ± 0.2 ^a^	5.9 ± 0.0 ^b,c^	0.1 ± 0.0 ^a,b^	16.2 ± 0.4 ^b^	3.1 ± 0.1 ^a,b^	7.9 ± 0.5 ^b^	34.3 ± 0.2 ^c^	-	-
M1	5.6 ± 0.1 ^a^	4.1 ± 0.0 ^a^	0.4 ± 0.0 ^c^	31.5 ± 0.2 ^c^	6.5 ± 0.2 ^b,c^	12.9 ± 0.0 ^c^	80.6 ± 1.2 ^d,e^	-	-
M2	18.1 ± 0.1 ^c^	3.9 ± 0.2 ^a^	1.2 ± 0.1 ^d^	39.0 ± 0.2 ^d^	25.6 ± 2.8 ^d^	32.1 ± 0.0 ^f^	82.2 ± 4.2 ^d,e^	-	-
M3	27.6 ± 1.3 ^d^	6.7 ± 0.4 ^c^	1.9 ± 0.0 ^e^	47.0 ± 1.3 ^e^	27.2 ± 2.1 ^d^	25.9 ± 0.5 ^e^	149.0 ± 7.0 ^f^	-	-
M4	30.0 ± 2.4 ^d^	5.4 ± 0.1 ^b^	2.0 ± 0.1 ^e^	56.0 ± 1.7 ^f^	5.1 ± 0.3 ^a,b^	16.9 ± 0.5 ^d^	87.9 ± 1.1 ^e^	-	-
M5	28.9 ± 1.2 ^d^	3.7 ± 0.4 ^a^	0.2 ± 0.0 ^b^	39.8 ± 2.5 ^d^	5.1 ± 0.3 ^a,b^	10.4 ± 2.2 ^b,c^	74.1 ± 3.5 ^d^	-	-
M6	38.9 ± 3.9 ^e^	10.6 ± 1.0 ^e^	N.D.	2.5 ± 0.1 ^a^	4.8 ± 0.9 ^c^	38.2 ± 2.3 ^g^	9.8 ± 0.7 ^a^	-	-
DM0	47.6 ± 0.9 ^C^	4.2 ± 0.2 ^A^	1.6 ± 0.1 ^C^	9.5 ± 0.2 ^B^	3.9 ± 0.2 ^C^	14.8 ± 0.2 ^C^	78.6 ± 8.2 ^D^	-	-
DM2	47.1 ± 0.9 ^C^	3.7 ± 0.5 ^A^	1.6 ± 0.1 ^C^	9.5 ± 0.5 ^B^	3.4 ± 0.1 ^B^	16.5 ± 0.6 ^D^	63.2 ± 5.2 ^C^	-	-
DM4	47.3 ± 0.0 ^C^	3.6 ± 0.5 ^A^	1.4 ± 0.0 ^B^	9.5 ± 0.1 ^B^	3.4 ± 0.0 ^B^	10.9 ± 0.1 ^B^	51.2 ± 3.5 ^B,C^	-	-
DM6	45.2 ± 0.1 ^B^	3.5 ± 0.2 ^A^	0.9 ± 0.0 ^A^	9.5 ± 0.5 ^B^	2.5 ± 0.1 ^A^	10.2 ± 0.1 ^B^	50.1 ± 0.9 ^B^	-	-
**W**	Chlorogenic Acid *^1^	***p*** **-Coumaric Acid**	**Ferulic Acid**	***p*** -Coumaric Acid *	Caffeic Acid *	***p*** -Coumaric Acid *^2^	**Quercetin-3-glucoside**	Luteolin *	Quercetin *
F	58.0 ± 2.7 ^g,B^	5.3 ± 1.3 ^d,B^	14.5 ± 1.8 ^d,B^	54.0 ± 0.6 ^f,B^	214.9 ± 1.6 ^h,C^	12.4 ± 0.6 ^e,B^	37.8 ± 1.1 ^c,B^	30.9 ± 0.1 ^d,D^	92.2 ± 2.5 ^e,B^
M0	36.1 ± 0.7 ^e^	5.0 ± 0.5 ^d^	15.3 ± 0.6 ^d^	34.7 ± 0.4 ^d^	174.6 ± 2.0 ^g^	7.9 ± 0.5 ^c^	55.7 ± 0.9 ^d^	48.0 ± 0.9 ^f^	69.0 ± 1.7 ^d^
M1	49.0 ± 0.6 ^f^	3.6 ± 0.1 ^c^	8.4 ± 0.3 ^c^	30.1 ± 0.2 ^c^	154.6 ± 1.0 ^f^	8.2 ± 0.1 ^c^	41.3 ± 0.8 ^c^	36.4 ± 0.6 ^e^	64.6 ± 0.9 ^d^
M2	22.3 ± 1.9 ^d^	1.4 ± 0.1 ^a,b^	5.0 ± 0.1 ^b^	10.6 ± 1.4 ^b^	52.5 ± 0.3 ^e^	3.0 ± 0.2 ^b^	23.1 ± 0.5 ^b^	24.2 ± 3.3 ^c^	29.7 ± 1.4 ^b^
M3	12.0 ± 0.7 ^c^	0.3 ± 0.1 ^a^	1.2 ± 0.3 ^a^	5.6 ± 0.3 ^a^	26.9 ± 0.1 ^c^	1.3 ± 0.2 ^a^	13.6 ± 0.3 ^a^	14.8 ± 0.3 ^b^	19.3 ± 1.4 ^a^
M4	5.2 ± 0.4 ^a^	1.0 ± 0.0 ^a,b^	1.7 ± 0.1 ^a^	10.5 ± 0.4 ^b^	8.0 ± 0.7 ^a^	10.5 ± 0.5 ^d^	23.7 ± 2.0 ^b^	4.9 ± 0.7 ^a^	45.2 ± 0.7 ^c^
M5	6.7 ± 0.2 ^a,b^	1.9 ± 0.1 ^b^	6.2 ± 0.6 ^b^	47.9 ± 0.5 ^e^	13.2 ± 0.2 ^b^	48.3 ± 0.7 ^f^	86.3 ± 0.6 ^e^	18.6 ± 0.2 ^b^	140.9 ± 0.8 ^f^
M6	8.1 ± 0.1 ^b^	1.1 ± 0.1 ^a,b^	6.5 ± 0.5 ^b^	58.1 ± 0.2 ^g^	30.9 ± 0.2 ^d^	58.2 ± 0.4 ^g^	104.8 ± 6.6 ^f^	25.6 ± 0.4 ^c^	168.6 ± 8.3 ^g^
DM0	2.5 ± 0.1 ^A^	3.5 ± 0.1 ^A^	7.9 ± 0.1 ^A^	27.4 ± 0.4 ^A^	142.8 ± 0.2 ^A,B^	6.7 ± 0.2 ^A^	43.2 ± 0.2 ^D^	10.3 ± 0.4 ^C^	63.4 ± 0.4 ^A^
DM2	2.1 ± 0.2 ^A^	3.3 ± 0.0 ^A^	7.3 ± 0.0 ^A^	26.3 ± 0.5 ^A^	143.3 ± 0.7 ^B^	6.5 ± 0.2 ^A^	41.7 ± 0.4 ^C^	7.3 ± 0.2 ^A,B^	61.2 ± 0.6 ^A^
DM4	2.1 ± 0.0 ^A^	3.2 ± 0.0 ^A^	7.0 ± 0.3 ^A^	26.4 ± 0.7 ^A^	141.5 ± 1.4 ^A,B^	6.2 ± 0.1 ^A^	36.5 ± 0.4 ^A^	6.8 ± 0.1 ^A^	62.6 ± 0.6 ^A^
DM6	2.0 ± 0.2 ^A^	3.4 ± 0.1 ^A^	8.3 ± 0.3 ^A^	27.1 ± 0.6 ^A^	140.3 ± 0.7 ^A^	6.5 ± 0.3 ^A^	42.9 ± 0.4 ^D^	8.1 ± 0.8 ^B^	63.6 ± 0.9 ^A^

Columns with different superscript letters and corresponding to the same byproduct and storage approach are significantly different (*p* < 0.05). Lowercase letters were used for fresh and frozen pulps forms, while uppercase letters were used for fresh and dry powders. * = derivative. *^1^ = derivative 1; *^2^ = derivative 2; N.D. = not detected.

**Table 4:** Main characteristics of polyphenols identified in the rocket, spinach and/or watercress byproducts.

Group of Phenolic Antioxidants	Phenolic Antioxidants	Chemical Formula	Retention Time (min)	λ max (nm)
	**Hydroxycinnamic acid**			
Phenolic acids	Chlorogenic acid	C_16_H_18_O_9_	27.2	326.0
	Caffeic acid	C_9_H_8_O_4_	31.0	323.6
	*p*-coumaric acid	C_9_H_8_O_3_	39.9	309.3
	Ferulic acid	C_10_H_10_O_4_	42.4	322.4
	Trans-ferulic acid	C_10_H_10_O_4_	42.6	322.4
	**Flavonols**			
	Quercetin-3-glucoside	C_15_H_10_O_7_	49.9	343.9
	**Flavones**			
Flavanoids	Luteolin-7-O-glucoside	C_15_H_10_O_6_	48.3	354.7
	Rutin	C_27_H_30_O_16_	49.8	353.5
	Apigenin	C_15_H_10_O_5_	53.7	268.0
	**Flavanols (cathecin)**			
	Epigallocatechin gallate	C_22_H_18_O_11_	29.4	276.1

## Data Availability

Not applicable.
